# The Association Between Fear of Crime, Life Satisfaction, and Health-Related Quality of Life in Non-Victimized Older Adults Aged 60–93 Years—Findings from the Swedish Good Aging in Skåne (GÅS) Population Based Study

**DOI:** 10.3390/ijerph23050641

**Published:** 2026-05-12

**Authors:** Emil Larsson, Sölve Elmståhl, Henrik Ekström

**Affiliations:** The Department of Clinical Sciences in Malmö, Division of Geriatric Medicine, Skåne University Hospital, Lund University, 205 02 Malmö, Sweden; em5718la-s@student.lu.se (E.L.); solve.elmstahl@med.lu.se (S.E.)

**Keywords:** fear of crime, health related quality of life, life satisfaction, older adults, population study

## Abstract

**Highlights:**

**Public health relevance—How does this work relate to a public health issue?**

- Fear of crime was common among older adults, with one-fifth of men and 50% of women reporting fear of becoming a crime victim when outdoors.
- Fear of crime is an individual as well as a societal problem expected to increase in line with the increase in the older population.

**Public health significance—Why is this work of significance to public health?**

- Fear of crime is a stress factor which may be associated with physical and mental illness.
- Fear of crime has been shown to be associated with a deterioration in physical and mental health-related quality of life and lower life satisfaction.

**Public health implications—What are the key implications or messages for practitioners, policy makers and/or researchers in public health?**

- Quality of life and the negative impact of fear of crime on the lives of older adults should be considered in the clinical setting, domestic services, and other healthcare contacts.
- Politicians and decision makers at both national and municipal levels should be informed about the extent of older adults’ fear of becoming a crime victim and that such fear may impact quality of life negatively. In addition to taking measures against crime itself, the design of the urban environment is important for creating safety.

**Abstract:**

Fear of crime (FOC) has been shown to be associated with negative physical and mental health effects, and older adults have been identified as a vulnerable group. As an individual as well as a societal problem, it is expected to increase in line with the growth of the older population. Nevertheless, the associations between FOC, life satisfaction (LS), and health-related quality of life (HRQoL) among non-victimized older adults are not well understood. Thus, the aim of the present study was to investigate whether levels of behavioral FOC in a sample of non-victimized older adults aged 60–93 years (mean age 69.6 years) were associated with HRQoL and LS. In this cross-sectional study a total of 5832 participants, representing both urban and rural areas, constituted the study sample. Associations between levels of behavioral FOC and LS as well as physical and mental HRQoL were examined in adjusted linear regression models. The results revealed that in those not previously exposed to violence or threats, FOC was associated with lower LS and poorer physical and mental HRQoL. When deterioration in HRQoL and LS in non-victimized older adults is discussed in a clinical setting, the possible link to FOC as an often unprovoked social stressor should be considered.

## 1. Introduction

Fear is a strong, unpleasant feeling and can have a positive function in terms of warning about an imminent danger. However, excessive fear with reactions such as breathing difficulties, heart problems, and anxiety, or a longer period of fear without a relevant reason, can negatively affect both physical and mental health [1]. Several common causes of fear among older adults have been described, such as the fear of illness, death, losing one’s life partner, not being allowed to remain in one’s own home, or being unable to cope financially [2]. Another fear among older adults that is not as commonly described and with equally serious social, psychological, and/or medical consequences is the fear of crime (FOC), i.e., the fear of being subjected to violence or the threat of violence. FOC has been described as multifaceted fear, including affective, cognitive, and behavioral dimensions, often measured by the frequency of the fear, perceived risk of victimization, and avoidant behaviors [2,3].

Fear of crime (FOC) has been reported to have several different causes on both an individual level and in a neighborhood context. The individual level causes include being a woman, older age, physical and mental illness, a lower educational level, and financial insufficiency. Causes in a neighborhood context are high crime rate, vandalism, and drug trafficking, as well as the design of the residential area, including lighting and the presence of surveillance cameras [4,5]. Older adults’ experience of being subjected to violence is a rational explanation for such fear, but when the fear of violence or the threat of violence occurs despite the absence of previous violent or threatening situations, it is called the FOC paradox. This means that many older adults feel afraid of becoming a victim of a crime even though they are statistically less likely to experience threats or violence [6]. A previous study by Köber et al. found no difference between younger and older adults in terms of the affective or cognitive component, only the behavioral component [7]. Most studies on FOC and the FOC paradox compare older adults as a group with younger adults. However, the older population is growing and becoming increasingly heterogeneous, making it important to compare different age subgroups among older adults.

The theoretical framework that we used in this study is how a society’s perceived crime subjectively and emotionally (affectively and cognitively) can lead to the FOC shaped by an individual’s previous experiences of victimization, environmental disorder, physical and psychological vulnerability, or adverse social integration [8] and whether the FOC in turn can affect the quality of life (QoL), either directly or via changed behavior. The relationship we have analyzed is whether FOC is expressed as a changed behavior, not daring to go out in the evening for fear of being exposed to violence or threats of violence, and whether this fear is associated with QoL adjusted for lifestyle, economic, medical, and social covariates.

The behavioral aspect of the FOC influences how people interact with their environment and communities, and may affect everyday life. It may become more difficult to implement collective solutions and collaborations within the local community. Not daring to go out in the evening can at an individual level lead to an increased degree of loneliness and social isolation and a risk of addictive behavior. If the behavior means that one feels compelled to protect oneself by installing surveillance cameras or alarms, it can entail financial costs. Not daring to be outdoors can lead to poorer health, either because one is less physically active or because one avoids seeking care. FOC has been linked to a range of negative health outcomes, including poorer mental health, limitations in physical function as well as in activities of daily living (ADL), poorer self-rated health, financial problems [9,10,11], and lower life satisfaction (LS) [12]. Less is known about the associations between the behavioral FOC and quality of life (QoL) among the different age cohorts of non-victimized older adults. Furthermore, studies on FOC and QoL do not consider different chronic diseases, which often have a significant impact on QoL in older adults.

Hence, this study aims to investigate the association between the behavioral FOC and QoL in a general population aged 60–93 years by examining three aspects of QoL, physical and mental HRQoL and global LS adjusted for demographic, socioeconomic, and health-related covariates.

## 2. Materials and Methods

### 2.1. Study Design and Participants

The participants in this cross-sectional study were drawn from the Good Aging in Skåne (GÅS) longitudinal general population-based study, which has been ongoing since 2001 and is part of the Swedish National Aging and Care (SNAC) survey [13]. The design of the GÅS study is described by Ekström et al. [14]. Individuals randomly selected from the national population register were invited by letter or phone. Informed consent was obtained from those who agreed to participate. In the present study, four waves comprising 11,562 individuals were invited to participate from 2001 to 2022. The first wave assessed from 2001 to 2004 (n = 5370) included nine age cohorts (60, 66, 72, 78, 81, 84, 87, 90, and 93 years); the second wave assessed between 2006 and 2012 (n = 2307) included two age cohorts (60 and 81 years); the third wave assessed during the period 2012–2016 (n = 2018) comprised two age cohorts (60 and 81 years); while the fourth wave assessed from 2017 to 2022 (n = 1867) included two age cohorts (60 and 81 years).

**Figure 1:**
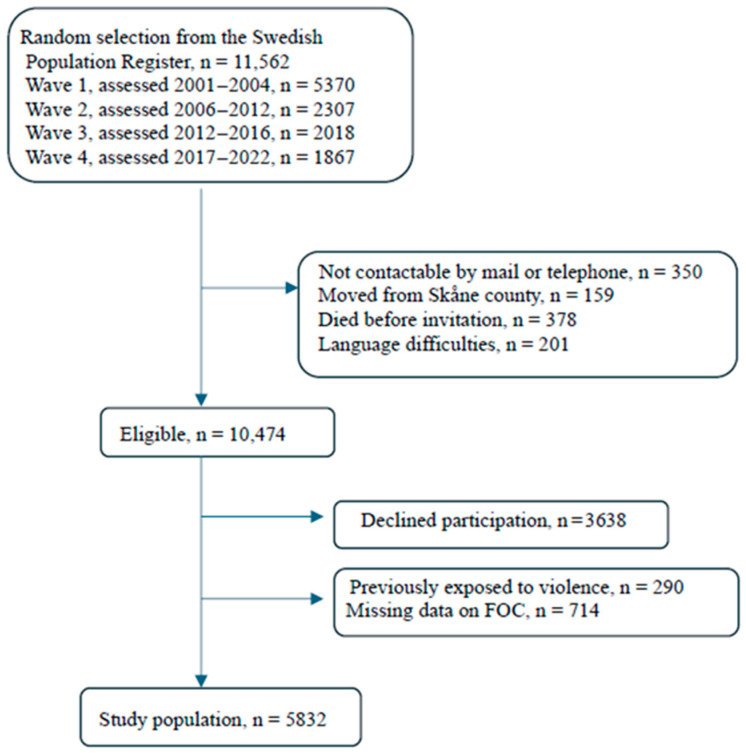
Flow diagram describing the inclusion of participants in the study.

Of the 11,562 GÅS informants invited to participate, 350 (3.0%) were not contactable, 159 (1.4%) had moved away from Skåne county, 201 (1.7%) had difficulty understanding Swedish, and 378 (3.3%) had died shortly before the invitation was sent and were therefore excluded. Out of the remaining 10, 474 eligible informants, 3638 (34.7%) declined participation, 714 (6.8%) were excluded due to missing data on FOC, as well as 290 (2.8%) who had previously been exposed to violence, which could trigger a true FOC. Finally, out of 10,474 eligible individuals, the study sample comprised 5832 (55.7%) participants, 3170 (54.4%) women and 2662 (45.6%) men (Figure 1).

Self-report questionnaires were used to obtain data on FOC, socio-demographic variables, lifestyle habits, and functional capacity. Trained assistants were on hand to help should any ambiguities arise. To assess cognitive status and depressive mood, the participants underwent a comprehensive, standardized psychological test conducted by a qualified psychologist. Previous or present diseases were confirmed or diagnosed in a medical examination performed by a physician. The examinations took place at the research clinic or in the participants’ homes due to impaired health or other reasons such as transport problems.

### 2.2. Assessment of Fear of Crime (FOC)

The FOC was assessed by a single-item question: “In the past year have you refrained from going out in the evening for fear of being assaulted, robbed, or molested?” Response alternatives were never, occasionally, quite often, often and very often. This question represents the behavioral aspect of FOC, as described by Greve et al. [3]. The responses quite often and often represented only 5.9% and 4.1% respectively and were therefore combined into a single category labeled often, resulting in a variable with four response options.

### 2.3. Assessment of Health-Related Quality of Life (HRQoL) and Life Satisfaction (LS)

HRQoL was assessed by the Short Form Health Survey (SF-12). The SF-12 (a short version of the SF-36 questionnaire), which is one of the most common instruments for evaluating HRQoL, includes 12 questions measuring HRQoL divided into two subscales. A physical component (PCS) comprises six questions covering the four domains of general health, physical functioning, physical role limitation, and bodily pain, as well as a mental component (MCS) covering the domains of role limitation, social functioning, vitality, and mental health. The score for both subscales ranges between 0 and 100 points and a higher score indicates higher HRQoL. The SF-12 has been evaluated for validity and reliability in several population studies and has been widely used internationally in both physical and mental health research [15,16].

LS was assessed using Neugarten’s quality of life scale (LSI-A) [17]. The LSI-A is a multidimensional instrument and consists of 20 attitude questions reflecting perceived LS in old age. The LSI-A covers 5 domains: Zest and mood tone reflect how LS is perceived at the present time, while the resolution and fortitude, positive self-concept, and congruence between the desired and achieved goals in life dimensions cover both the past and thoughts about the future and life in general. Response alternatives to each of the 20 questions are disagree (0 points), doubtful (1 point), and agree (2 points), and a high score (range 0–40 points) indicates a higher LS. Internal consistency reliability and validity were established by Neugarten [17], and the normative values for the general older Swedish population have been previously established by the present researchers [18].

### 2.4. Covariates: Socio-Demographics and Medical History

Several covariates that may confound the relationship between FOC, HRQoL, and LS were adjusted for in the regression models. Socio-demographic data included age, sex, education, cohabiting status, place of residence, financial status, alcohol habits, smoking habits, physical activity, ADL, cognitive status, and attitude towards older adults.

Level of education was trichotomized into elementary school (9 years of compulsory studies), secondary school (3–4 years of optional studies), and college/university (more than 1 year of college/university studies).

Cohabiting status was dichotomized into cohabiting and living alone. Financial status was dichotomized as poor or good based on whether the participants answered yes or no to the question “Have you had difficulties making ends meet when it comes to running expenses during the past year?” Alcohol habits were categorized as never, alcohol consumption 1–4 times a month, twice a week, or more often.

ADL was assessed by the Katz ADL index, measuring personal functioning capacity in six activities: bathing, dressing and undressing, toileting, continence, movement, and food intake. Participants were dichotomized as functionally independent (ADL 0) or functionally dependent if dependent in one or more activities (ADL 1–6) [19]. Participants’ perceptions of attitude toward older people in society were assessed using very positive, positive, neither positive nor negative, negative, and very negative. These responses were subsequently recategorized into positive, neutral, and negative. The Katz ADL index has shown validity and reliability in the Swedish setting [20].

Depressive mood was measured by the Montgomery-Åsberg Depression Rating Scale (MADRS), a subscale of the Comprehensive Psychopathological Rating Scale (CPRS) [21]. The MADRS comprises 10 questions, including items pertaining to anxiety, lack of initiative, reduced emotional involvement, and life-threatening and suicidal thoughts. Each question is rated from 0 to 6 points, and the scale ranges from 0 to 60 points. A score > 6 points indicates a depressive mood [22]. The test was conducted as a structured interview by a psychologist. The MADRS has been reported to be a reliable instrument for detecting depression in non-demented older adults [23].

Cognitive impairment was assessed by the Mini Mental State Examination (MMSE), measuring global cognitive function. The scale ranges between 0 and 30 points, and cognitive impairment was set at <25 points [24].

Diseases were evaluated by the study physician and grouped under the categories of heart disease (myocardial infarction, angina pectoris, arrhythmia), hypertension, cerebrovascular disease (stroke, transient ischemic attack, reversible ischemic neurologic deficit), endocrine disease (diabetes type 1 or 2, thyroid disease), pulmonary disease (asthma, chronic obstructive pulmonary disease, tuberculosis), musculoskeletal disease (osteoporosis, arthrosis, inflammatory joint disease, hip fracture), and cancer (any type of malignant tumor). The number of morbidity categories was categorized as 0, 1, 2, or ≥3 [25].

### 2.5. Statistical Analysis

Statistical significance of the differences in proportions regarding age, sex, education, cohabiting status, place of residence, financial status, alcohol habits, smoking habits, physical activity, ADL, cognitive status, attitude toward older adults, depressive mood, and disease categories in relation to FOC levels was tested with the Chi-squared (χ^2^) test. Differences in LSI-A, SF-12, PCS, and MCS sum scores in relation to FOC levels were tested with the Kruskal–Wallis test.

To evaluate the possible associations between the levels of the FOC and the LSI-A, SF-12, PCS, and MCS scores, adjusted standard multiple linear regressions were constructed and regression coefficients calculated. All of the presented independent variables and the dependent variable FOC were simultaneously entered into the regression models, and both the independent variables and the dependent variable FOC were used as dummies.

Multicollinearity was tested for in all of the regression models, where none of the included variables had a variance inflation factor > 5.0 [26]. The multiple linear regression models were further tested for the assumptions of normality, linearity, and homoscedasticity. In each multiple regression model, normality was controlled for by inspecting the histograms of the residuals and linearity by checking the scatter plots (standardized predicted values vs. standardized residuals), but no unacceptable deviations were noted [27]. To address heteroscedasticity, the results of the regression models are presented with calculated robust standard errors (HC3). Sensitivity analyses with multiple imputations of missing data were conducted. Five imputed datasets were generated using the fully conditional specification method (FCS). The imputed model included all of the variables used in the regression analysis. The parameter estimates were pooled according to Rubin’s rules (Table A1, Table A2 and Table A3).

A *p*-value of <0.05 was considered to indicate statistical significance. All analyses were conducted using SPSS^®^ version 30 and 31 (IBM SPSS Statistics for Windows).

## 3. Results

### 3.1. The Study Sample

The study population comprised 5832 participants (Table 1). Additionally, 54.4% were women and 45.6% men, and their mean age was 69.6 years (SD 10.3); 34.7% reported FOC at least occasionally (47.7% of women and 19.3% of men), 26.1% had attended university, 39.4% lived alone, and 86.7% lived in an urban environment. In addition, 12.3% were dependent on at least one ADL, and 12.4% had a suspected cognitive impairment. The most common disease category was musculoskeletal diseases reported by 36.7%, followed by hypertension (32.6%) and heart disease (21.0%) (Table 1).

To summarize the outcome of the studied independent variables in relation to FOC levels, there was a greater proportion of FOC among women, those aged 80 years and older, those living alone, those with a lower educational level or financial problems, those with a cognitive impairment, those who were ADL dependent, and those affected by illness. Among those with more frequent drinking habits, the proportion of FOC was lower compared to those who drank alcohol less frequently. The proportion who never hesitated to go out in the evening for fear of being harassed varied between assessment periods, with the highest proportion reported among those included in the third round between 2020 and 2016. (Table 2 and Table 3).

For the HRQoL and LS outcome variables, the results were significantly poorer in all three scales, with a tendency toward a higher level of FOC, i.e., the more often one avoided going out for fear of being assaulted, robbed, or molested, the lower the SF-12 (PCS, MCS) and the LSI-A scores (Table 3).

Fear of crime was based on the following question: “In the past year have you refrained from going out in the evening for fear of being assaulted, robbed, or molested?” with the response alternatives: never, occasionally, often, and very often.

### 3.2. HRQoL and LSI-A

In the multiple linear regression model, higher FOC levels were associated with a decrease in SF-12 (PCS, MCS) and LSI-A scores, indicating poorer HRQoL and lower LS. Those with the most frequent FOC reported significantly lower scores on the LSI-A (Table 4) and SF-12 (PCS, MCS) (Table 5 and Table 6) compared to those with no FOC or FOC only occasionally.

When analyzing the covariates, we noted that participants aged 80 years and older reported lower scores on the PCS and LS scales but higher scores on the MCS in the regression models, suggesting better mental health in this group. A negative perceived attitude toward older people was associated with lower LS and MCS scores, while a positive attitude was linked to higher scores across all scales. Financial difficulties, current or former smoking status, dependence in ADL, and ≥3 morbidities were all associated with lower LS, PCS, and MCS scores. Additionally, the regression models revealed that female sex was related to lower PCS and MCS scores. Depression was strongly associated with both LS and PCS scores. Wave 3 included in the 2012–2016 study was related to lower MCS scores and higher LS scores.

## 4. Discussion

This cross-sectional study aimed to investigate whether levels of behavioral FOC in a sample of non-victimized older adults, mean age 69.6 years, were associated with HRQoL and LS. Although studies from other countries have shown such relationships [11,12], little has been published on the behavioral component of FOC or differences between age groups among older adults or adjusted for comorbidities. We found that FOC levels were significantly associated with both lower SF-12 (PCS and MCS) and lower LSI-A scores after adjusting for various socio-demographic and health variables, with a tendency toward lower scores with increasing FOC levels.

Although we have shown statistical relationships between FOC and QoL, the question is whether these are relevant and whether the results mean anything in practice. In general, it can be said that it depends on what the study population looks like and in what context the survey is conducted. However, if one looks at life satisfaction as a global measure of well-being, the norm values for a general Swedish population 60 years and older have been calculated for the LSA-A to be 28 points with a standard deviation of 7 points. The adjusted difference of 2 points, between the groups of never being influenced by FOC in their behavior and very often doing so, is not insignificant [18]. In addition to a worse mood and deteriorating mental and physical health and a reduced social engagement of the individual [28], lower life satisfaction can indirectly be a cause for greater FOC. That is, the inverse relationship we have tried to highlight, and a vicious circle between FOC and QoL can become the case and possibly contribute even more to the negative societal consequences, for example, an increased burden on healthcare and social care, both in terms of work effort and costs [29].

As mentioned above, the FOC paradox [6], i.e., those with the strongest fear of becoming a victim of crime are those least affected by violence or threats of violence was in line with the descriptive findings from this study. Women and participants aged 70 and older reported significantly higher levels of FOC compared to men and participants aged 60 [30,31]. However, the FOC paradox has been questioned, based on the possibility that the FOC experienced by women and older adults is not a paradox but a reflection of the complex factors that shape the perceptions of danger [31]. FOC can to some extent be explained by the fact that older adults perceive themselves as physically, mentally, or socially weaker and thus feel more vulnerable [32]. The latter may, for example, be due to experiencing one’s home environment as frightening or threatening because of vandalism or neighbors with whom one does not get along. The almost daily mass media reporting of violent crime and fraud in which older people are victims can also create fears about crime in general [33], although television and newspapers often convey a false picture, where serious violent crimes are overrepresented.

In the case of Sweden, young people’s involvement in serious criminality has increased in recent decades [34]; hence, depictions of crime victims and an unsafe society have become increasingly common in the daily press, leading to the fear being described as a natural response [35]. These factors could influence not only FOC but also a person’s perception of the present and future, which could impact LSI-A scores.

It should be noted that the question we used to operationalize FOC concerned whether people would refrain from going out in the evening due to fear of being subjected to violence or harassment. Therefore, participants responded to the fear of being harassed and its consequences and not FOC per se [36]. It can be difficult to distinguish between FOC per se and the fear of its consequences. However, in the present study this distinction is not crucial. For most participants behavioral FOC probably involves both, and there is no reason to believe that one might have a greater impact on LS and HRQoL compared to the other.

This study primarily investigated whether FOC was associated with LS and HRQoL. In theory, FOC can be considered a stressor that has a direct impact on health, as studies have demonstrated that the number of stressful events experienced during the previous year is inversely related to both mental and physical HRQoL among older adults [37]. Furthermore, the consequences of FOC, which in the worst-case scenario can resemble a phobia of violence or threats of violence, have been reported to involve social limitations [32]. In some studies the behavioral component has been divided into four categories: avoidance behavior, protective behavior, lifestyle adjustments, and participation in collective activities [38]. For example, people may avoid going out or only choose certain times and places to visit and stop participating in previous social activities or using various community services [32], which in turn can lead to social isolation or loneliness. The reverse relationship is plausible, i.e., that lower QoL leads to greater FOC. People who for several reasons, including medical, financial, or social factors, may perceive their QoL to be impaired might consider themselves more vulnerable and therefore have a greater fear of being exposed to violence or threats of violence [39]. FOC may increase anxiety or depressive mood, which in turn may lead to a greater tendency to be vulnerable to thoughts about crime and victimization. In addition, there is also a possibility that many of the negative effects caused by FOC are themselves risk factors for FOC, i.e., the consequences of FOC will reinforce already established risk factors, leading to a vicious circle with an ever greater FOC.

A relevant question is whether the included co-variates (risk factors) were correctly chosen [39]. We have previously reported the factors related to QoL in both cross-sectional and longitudinal studies from the GÅS study [40,41]. We have done our best to select relevant co-variates that can be associated with LS and HRQoL, i.e., sex, age, education, cohabiting status, financial status, and alcohol and smoking habits in addition to a geriatric perspective that includes functioning (ADL), depressive mood, and illnesses common in older adults, which can increase the feelings of vulnerability to violence or threats of violence [42].

The prevalence of FOC was much higher among women, a difference that has been discussed in previous studies [43,44]. Pain et al. argued that FOC in general is not overestimated among women but rather underestimated among men [44]. A plausible explanation for this is that men do not find it socially desirable or masculine to report being victims of crime [45]. At the same time, violence against women often remains unreported, with a high percentage of women not reporting sexual assault due to a sense of guilt and embarrassment [43,46]. Regarding age, even though FOC seems to increase with age and is associated with lower LS and HRQoL, the age of over 80 years was associated with a higher MCS score in the regression model. Compared to PCS, MCS seems to remain more stable when aging [47,48]. Greve et al. [3] found that the ability of older people to adjust to age by means of setting flexible goals decreased FOC while at the same time reducing the negative effects of FOC on mental health, something that could explain these results.

We included perceived attitude toward older adults in society as a covariate, which—to the authors’ knowledge—has not been previously explored. This was of interest given studies indicating that stereotypes of older adults have become more negative in recent years [49]. Other covariates such as education, financial difficulties, physical activity, and dependence in ADL were also related to lower HRQoL and LS, similar to the results that have been reported in other studies [50,51]. However, as FOC was the main independent variable of interest, a more detailed discussion of these associations is beyond the scope of the study.

### Strengths and Limitations

A strength of this study is the large study population representing both urban and rural environments, different living arrangements, and socio-economic factors. Participants were randomly selected. To reduce selection bias, home visits or phone interviews were conducted when participants were unable to travel to the research facility and assistance was provided for participants with, for example, hearing or vision impairment. Our data enabled us to exclude those who had been victims of crime; thus, we only included those who reported FOC without being a previous crime victim. Another strength is that this is one of the first studies—if not the first—to examine the relationship between FOC and QoL adjusted for chronic diseases, i.e., one of the first to add a medical perspective to the discussion about FOC. Nevertheless, one should be aware that people who choose to participate in epidemiological studies are healthier and in a more socio-economically favorable position compared to non-participants [52]. This fact might reduce the generalizability of our study. It should also be mentioned that the uneven age distribution with only 8.8% participants aged 70–79 is another shortcoming that might reduce the generalizability of this study. Another possible limitation is that we categorized continuous variables in our regression models, which often results in a loss of information. The reason was that we wanted to make it easier to interpret the results. It is often easier to discuss, for example, what a dependency in ADL or a depressive state means in relation to FOC than to interpret what a change in a continuous scale means.

At the same time, despite several relevant explanatory variables being included in the regression models, the R^2^ value (the coefficient of determination representing the proportion of variance in a dependent variable explained by the independent variables) is small in all regression models, especially in the model with SF-12 MCS as the outcome variable. In practice, this can be interpreted as FOC having modest significance in explaining the association to life satisfaction or health-related quality of life in older adults. However, it should be said that the cross-sectional design of this study precludes the evaluation of the direction of the associations of FOC and LS and HRQoL.

In the study, we explored differences in FOC based on whether the participants lived in an urban or rural environment. However, we lacked data on the design of the urban environment itself that may be significant, such as parks, pedestrian tunnels, or surveillance cameras, which may affect FOC [53]. Nor did we have access to data on the current crime in the residential areas in question, which may also affect FOC.

As mentioned in the introduction, FOC can be looked at from different perspectives [3]. In this study, a single-item question was used to assess the behavioral aspect of FOC. It is not known whether the other aspects of FOC, i.e., participants’ frequency of fear (the affective component) and perceived risk of victimization (the cognitive component), have the same associations with LS and HRQoL as the behavioral component [3].

Only participants who had complete data on all variables were included in the regression models, which may lead to the detection of false associations. To check whether the attrition affected the overall result, sensitivity analyses with multiple imputations were performed in all regression models. Comparisons of the results before and after multiple imputations showed minor differences. The overall results remained, see Table A1, Table A2 and Table A3, page.

Not daring to go outdoors due to fear of becoming a victim of crime was the starting point of the study. At the same time, it should be remembered that there are many more reasons why people avoid going out. We have taken some into account in our analyses, such as ADL, cognition, depressive mood, and illness. Important reasons that have not been included are, for example, characteristics of the neighborhood environment or mobility limitations.

Participants were recruited between 2001 and 2022, and we cannot rule out the presence of cohort effects in the study population. It is possible that the prevalence of FOC has changed over time due to local and global factors, for example, an increase in crime [34] or the COVID-19 pandemic. Analyzing historical social or societal causes that can explain FOC in the different cohorts (waves 1–4) such as crime rate, political decisions aimed at reducing crime, newly started associations for the elderly in the area, how the mass media reports on crime, or the redevelopment of run-down areas is beyond the scope of this cross-sectional study, but we can conclude that adjusted for the assessment periods, the associations between FOC and SF-12 PCS, MCS, and LSI-A remain. Further research with a different study design is warranted to identify a causal relationship between FOC and health outcomes.

Another limitation, but something that will constitute the subject of future studies, is that we did not study FOC in relation to fear of the consequences of harassment, such as physical and psychological harm or fear of the destruction of personal belongings [36], or fear of loved ones being subjected to violence or threats of violence [54].

## 5. Conclusions

This study has shown that FOC in non-victimized older adults is associated with reduced LS and HRQoL. At an individual level, the possibility of FOC and its negative impact on QoL should be considered in the clinical setting, domestic services, and other healthcare contacts, as it is often an unfounded but experienced stressor that negatively influences the QoL of older adults. From a societal perspective, politicians and decision makers at both national and municipal levels should be informed about the extent of FOC among older people and that such fear may be associated with reduced QoL. In addition to taking measures to reduce crime, they should consider what can be done in the design of the urban environment to create security so that older people do not avoid spending time outdoors.

## Figures and Tables

**Table 1:** Description of study sample, n = 5832.

Category Variable	Groups	Number (%)	Missing (%)
Fear of crime (FOC)	Never	3806 (65.3)	0 (0)
	Occasionally	1183 (20.3)	
	Often	585 (10.0)	
	Very often	258 (4.4)	
Sex	Male	2662 (45.6)	0 (0)
	Female	3170 (54.4)	
Age group	60–69	3486 (59.8)	0 (0)
	70–79	511 (8.8)	
	80+	1835 (31.5)	
	Mean (SD)	69.6 (10.3)	
Cohabiting status	Cohabitating	3531 (60.5)	6 (0.1)
	Living alone	2295 (39.4)	
Education	Primary	2454 (42.1)	28 (0.5)
	High School	1827 (31.3)	
	University	1523 (26.1)	
Place of residence	Rural	769 (13.2)	8 (0.1)
	Urban	5055 (86.7)	
Financial difficulties	Yes	279 (4.8)	18 (0.3)
Alcohol habits	Never	1115 (19.1)	22 (0.4)
	Monthly	3288 (56.4)	
	Weekly	1407 (24.1)	
Smoking habits	Never	2466 (42.3)	10 (0.2)
	Quit smoking	2450 (42.0)	
	Current smoker	906 (15.5)	
Physical activity	Sedentary	1084 (18.6)	22 (0.4)
	Light	2784 (47.7)	
	Moderate to strenuous	1942 (33.3)	
Perceived attitude towards older adults	Positive	1534 (26.3)	10 (0.2)
	Neutral	2649 (45.4)	
	Negative	1639 (28.1)	
Activities of daily living (ADL)	Dependent	719 (12.3)	28 (0.5)
Cognitive impairment	MMSE 0–24	721 (12.4)	221 (3.8)
Depressive mood	MADRS > 6	744 (12.8)	389 (6.7)
Physical morbidities	Heart disease	1223 (21.0)	113 (1.9)
	Hypertension	1900 (32.6)	116 (2.0)
	Cerebrovascular disease	541 (9.3)	115 (2.0)
	Endocrine disease	997 (17.1)	110 (1.9)
	Pulmonary disease	715 (12.3)	111 (1.9)
	Musculoskeletal disease	2135 (36.7)	151 (2.6)
	Cancer	900 (15.4)	111 (1.9)
Number of morbidities	0	1506 (25.8)	103 (1.8)
	1	1760 (30.2)	
	2	1273 (21.8)	
	≥3	1190 (20.4)	
Quality of life			
Life satisfaction index-A (LSI-A)	Median (IQR)	29 (24–33)	95 (1.6)
SF-12 Physical Component Summary	Median (IQR)	49.8 (38.1–54.8)	105 (1.8)
SF-12 Mental component Summary	Median (IQR)	57.2 (51.2–59.8)	105 (1.8)

**Table 2:** Characteristics of the study sample. Socio-demographic, lifestyle, and time period of inclusion variables in relation to fear of crime. Differences in proportion were tested with the Chi squared (χ^2^) test, n = 5832.

Fear of Crime							
Socio-Demographic and Lifestyle Variables	Never n (%)	Occasionally n (%)	Often n (%)	Very Often n (%)	*p*- Value	Total n (%)	Missing n (%)
Sex							
Men	2149 (80.7)	375 (14.1)	114 (4.3)	24 (0.9)	<0.001	2662 (45.6)	0 (0)
Women	1657 (52.3)	808 (25.5)	471 (14.9)	234 (7.4)		3170 (54.4)	
Age group							
60–69	2546 (73.0)	643 (18.4)	226 (6.5)	71 (2.0)	<0.001	3486 (59.8)	0 (0)
70–79	275 (53.8)	133 (26.0)	73 (14.3)	30 (5.9)		511 (8.8)	
80+	985 (53.7)	407 (22.2)	286 (15.6)	157 (8.6)		1835 (31.5)	
Cohabiting status							
Cohabiting	2504 (70.9)	674 (19.1)	271 (7.7)	82 (2.3)	<0.001	3531 (60.6)	6 (0.1)
Living alone	1298 (56.6)	508 (22.1)	313 (13.6)	176 (7.7)		2295 (39.4)	
Education							
Elementary school	1417 (57.7)	517 (21.1)	356 (14.5)	164 (6.7)	<0.001	2454 (42.3)	28 (0.5)
High school	1225 (67.0)	383 (21.0)	154 (8.4)	65 (3.6)		1827 (31.5)	
University	1147 (75.3)	278 (18.3)	71 (4.7)	27 (1.8)		1523 (26,2)	
Place of residence							
Rural	615 (80.0)	106 (13.8)	34 (4.4)	14 (1.8)	<0.001	769 (13.2)	8 (0.1)
Urban	3185 (63.0)	1076 (21.3)	550 (10.9)	244 (4.8)		5055 (86.8)	
Financial difficulties							
Yes	160 (57.3)	65 (23.3)	38 (13.6)	16 (5.7)	0.029	279 (4.8)	18 (0.3)
No	3633 (65.6)	1116 (20.2)	544 (9.8)	242 (4.4)		5535 (95.2)	
Alcohol habits							
Never	607 (54.4)	226 (20.3)	181 (16.2)	101 (9.1)	<0.001	1115 (19.2)	22 (0.4)
1–4 times/month	2132 (64.8)	697 (21.2)	327 (9.9)	132 (4.0)		3288 (56.6)	
≥2 times/week	1057 (75.1)	255 (18.1)	71 (5.0)	24 (1.7)		1407 (24.2)	
Smoking habits							
Non-smoker	1520 (61.6)	513 (20.8)	294 (11.9)	139 (5.6)	<0.001	2466 (42.4)	10 (0.2)
Former smoker	1684 (68.7)	488 (19.9)	205 (8.4)	73 (3.0)		2450 (42.1)	
Current smoker	595 (65.7)	181 (20.0)	85 (9.4)	45 (5.0)		906 (15.6)	
Physical activity							
Sedentary	671 (61.9)	175 (16.1)	154 (14.2)	84 (7.7)	<0.001	1084 (18.7)	22 (0.4)
Lighter	1726 (62.0)	645 (23.2)	294 (10.6)	119 (4.3)		2784 (47.9)	
Moderate/strenuous	1395 (71.8)	360 (18.5)	133 (6.8)	54 (2.8)		1942 (33.4)	
Perceived attitude towards older adults							
Positive	1118 (72.9)	255 (16.6)	111 (7.2)	50 (3.3)	<0.001	1534 (26.3)	10 (0.2)
Neutral	1732 (65.4)	552 (20.8)	268 (10.1)	97 (3.7)		2649 (45.5)	
Negative	948 (57.8)	375 (22.9)	205 (12.5)	111 (6.8)		1639 (28.2)	
Time period of inclusion							
Wave 1, 2001–2004	1538 (60.2)	529 (20.7)	319 (12.5)	167 (6.5)	<0.001	2553 (43.8)	0 (0)
Wave 2, 2006–2012	840 (66.1)	286 (22.5)	111 (8.7)	34 (2.7)		1271 (21.8)	
Wave 3, 2012–2016	862 (75.3)	184 (16.1)	78 (6.8)	21 (1.8)		1145 (19.6)	
Wave 4, 2017–2022	566 (65.6)	184 (21.3)	77 (8.9)	36 (4.2)		863 (14.8)	

**Table 3:** Characteristics of the study sample. Health-related and quality of life variables in relation to fear of crime. Differences in proportions were tested with the Chi squared (χ^2^) test, n = 5832.

		Fear of Crime					
Health-Related Variables	Never n (%)	Occasionally n (%)	Often n (%)	Very Often n (%)	*p*- Value	Total n (%)	Missing n (%)
ADL status							
Independent	3392 (66.7)	1008 (19.8)	490 (9.6)	195 (3.8)	<0.001	5085 (87.6)	28 (0.5)
Dependent	395 (54.9)	170 (23.6)	93 (12.9)	61 (8.5)		719 (12.4)	
Cognitive functioning, MMSE							
0–24 p, impaired	399 (55.3)	154 (21.4)	117 (16.2)	51 (7.1)	<0.001	721 (12.8)	221 (3.8)
25–30 p, not impaired	3248 (66.4)	1002 (20.5)	443 (9.1)	197 (4.0)		4890 (87.2)	
Depressive mood							
Yes	376 (50.5)	164 (22.0)	138 (18.5)	66 (8.9)	<0.001	744 (13.7)	389 (6.7)
No	3182 (67.7)	951 (20.2)	400 (8.5)	166 (3.5)		4699 (86.3)	
Number of morbidities							
0	1133 (75.2)	250 (16.6)	88 (5.8)	35 (2.3)	<0.001	1560 (26.2)	103 (1.8)
1	1157 (65.7)	360 (20.5)	178 (10.1)	65 (3.7)		1760 (30.7)	
2	777 (61.0)	291 (22.9)	142 (11.2)	63 (4.9)		1273 (22.2)	
≥3	667 (56.1)	265 (22.3)	167 (14.0)	91 (7.6)		1190 (20.8)	
Quality of life							
LSI-A ^1^, median (IQR)	30 (25–34)	28 (23–32)	26 (20–30)	23 (18–29)	<0.001		95 (1.6)
SF-12 ^2^, median (IQR)							
PCS	51.6 (42.6–55.3)	48.6 (36.7–53.8)	42.7 (32.6–51.4)	39.7 (28.2–49.4)	<0.001		105 (1.8)
MCS	57.8 (53.6–60.0)	56.0 (50.1–59.4)	54.0 (43.1–58.6)	54.1 (42.8–59.6)	<0.001		105 (1.8)

^1^ Life satisfaction index-A, ^2^ short form 12, Physical Component Summary (PCS), and mental component summary (MCS).

**Table 4:** Multiple linear regression model with Neugarten’s LSI-A ^1^ score as the dependent variable, n = 5373 (92.1%).

Variable (Reference Category)		B	Robust Std. Error	95% CI	*p*-Value
FOC (never)	Occasionally	−0.54	0.21	−0.96/−0.12	0.011
	Often	−1.54	0.32	−2.16/−0.92	<0.001
	Very often	−2.05	0.47	−2.97/−1.13	<0.001
Sex (male)	Female	0.27	0.18	−0.08/0.62	0.131
Age group (60–69)	70–79	−0.25	0.32	−0.88/0.38	0.434
	80+	−0.97	0.23	−1.42/−0.52	<0.001
Cohabitating status (cohabitating)	Living alone	−1.95	0.18	−2.31/−1.59	<0.001
Education (primary)	High School	0.39	0.20	−0.01/0.79	0.051
	University	0.86	0.22	0.44/1.29	<0.001
Place of residence (rural)	Urban	−0.08	0.23	−0.53/0.37	0.736
Financial difficulties (no)	Yes	−2.75	0.46	−3.65/−1.85	<0.001
Alcohol consumption (never)	Monthly	0.30	0.24	−0.18/0.77	0.222
	Weekly	0.90	0.29	0.34/1.47	0.002
Smoking (never)	Previous smoker	−0.57	0.18	−0.93/−0.21	0.002
	Current smoker	−0.70	0.25	−1.19/−0.21	0.005
Physical activity (sedentary)	Light	1.50	0.25	1.01/1.99	<0.001
	Moderate/strenuous	2.38	0.27	1.86/2.91	<0.001
Attitude towards older adults (neutral)	Positive	1.65	0.19	1.27/2.03	<0.001
	Negative	−1.14	0.23	−1.54/−0.75	<0.001
ADL (independent)	Dependence in ADL	−1.63	0.29	−2.20/−1.06	<0.001
Cognitive functioning (MMSE ≤ 24)	MMSE ≥ 25	1.05	0.28	0.49/1.61	<0.001
Depressive mood (no)	Yes	−5.66	0.29	−6.22/−5.10	<0.001
Number of morbidities (0)	1	−0.07	0.21	−0.47/0.34	0.740
	2	−0.83	0.24	−1.30/−0.37	<0.001
	≥3	−1.12	0.29	−1.66/−0.59	<0.001
Time period of inclusion, years					
(Wave 1, 2001–2004)	Wave 2, 2006–2012	0.44	0.22	0.03/0.88	0.049
	Wave 3, 2012–2016	0.99	0.23	0.54/1.44	<0.001
	Wave 4, 2017–2022	0.35	0.27	−0.18/0.88	0.191
R^2^ = 0.285					

^1^ Neugarten’s Life Satisfaction Index (LSI-A).

**Table 5:** Multiple linear regression model with the SF-12 PCS ^1^ score as the dependent variable, n = 5367 (92.2%).

Variable (Reference Category)		B	Robust Std. Error	95% CI	*p*-Value
FOC (never)	Occasionally	−0.93	0.25	−1.42/−0.44	<0.001
	Often	−1.74	0.36	−2.45/−1.04	<0.001
	Very often	−2.43	0.58	−3.56/−1.30	<0.001
Sex (male)	Female	−0.81	0.20	−1.20/−0.42	<0.001
Age group (60–69)	70–79	−0.27	0.36	−0.98/0.45	0.463
	80+	−2.03	0.27	−2.56/−1.49	<0.001
Cohabitating status (cohabitating)	Living alone	−0.43	0.21	−0.84/−0.02	0.039
Education (primary)	High School	0.09	0.23	−0.36/0.54	0.693
	University	0.79	0.25	0.31/1.28	0.001
Place of residence (rural)	Urban	0.99	0.27	0.46/1.52	<0.001
Financial difficulties (no)	Yes	−1.49	0.52	−2.52/−0.47	0.004
Alcohol consumption (never)	Monthly	1.10	0.30	0.52/1.68	<0.001
	Weekly	1.80	0.37	1.14/2.46	<0.001
Smoking (never)	Previous smoker	−0.76	0.20	−1.17/−0.36	<0.001
	Current smoker	−1.28	0.28	−1.84/−0.72	<0.001
Physical activity (sedentary)	Light	3.63	0.32	3.00/4.25	<0.001
	Moderate/strenuous	5.43	0.33	4.78/6.08	<0.001
Attitude toward older adults (neutral)	Positive	0.71	0.22	0.27/1.14	0.002
	Negative	−0.35	0.23	−0.80/0.09	0.120
ADL (independent)	Dependence in ADL	−2.88	0.34	−3.54/−2.22	<0.001
Cognitive functioning (MMSE ≤ 24)	MMSE ≥ 25	1.31	0.34	0.64/1.97	<0.001
Depressive mood (no)	Yes	−3.08	0.32	−3.70/−2.45	<0.001
Number of morbidities (0)	1	−2.06	0.22	−2.45/−1.63	<0.001
	2	−3.82	0.27	−4.34/−3.30	<0.001
	≥3	−5.79	0.31	−6.40/−5.18	<0.001
Time period of inclusion, years					
(Wave 1, 2001–2004)	Wave 2, 2006–2012	0.18	0.24	−0.28/0.65	0.440
	Wave 3, 2012–2016	−0.13	0.29	−0.70/0.44	0.651
	Wave 4, 2017–2022	−0.21	0.30	−0.79/0.38	0.487
R^2^ = 0.335					

^1^ Physical Component Summary (PCS) from the 12-item Short Form Survey (SF-12).

**Table 6:** Multiple linear regression model with the SF-12 MCS ^1^ score as the dependent variable, n = 5519 (94.6%).

Variable (Reference Category)		B	Robust Std. Error	95% CI	*p*-Value
FOC (never)	Occasionally	−1.21	0.22	−1.63/−0.78	<0.001
	Often	−1.89	0.32	−2.54/−1.26	<0.001
	Very often	−2.00	0.49	−2.95/−1.04	<0.001
Sex (male)	Female	−0.51	0.19	−0.86/−0.17	<0.001
Age group (60–69)	70–79	1.00	0.29	0.44/1.57	0.003
	80+	0.91	0.23	0.46/1.36	<0.001
Cohabitating status (cohabitating)	Living alone	−0.70	0.18	−1.05/−0.34	<0.001
Education (primary)	High School	−0.46	0.19	−0.84/−0.08	0.019
	University	−0.75	0.21	−1.16/−0.34	<0.001
Place of residence (rural)	Urban	−0.08	0.23	−0.54/0.38	0.728
Financial difficulties (no)	Yes	−2.21	0.50	−3.20/−1.22	<0.001
Alcohol consumption (never)	Monthly	0.19	0.25	−0.30/0.67	0.448
	Weekly	0.13	0.29	−0.43/0.70	0.648
Smoking (never)	Previous smoker	−0.07	0.18	−0.42/0.29	0.704
	Current smoker	−0.36	0.25	−0.85/0.14	0.161
Physical activity (sedentary)	Light	1.10	0.26	0.56/1.62	<0.001
	Moderate/strenuous	1.26	0.28	0.71/1.82	<0.001
Attitude toward older adults (neutral)	Positive	0.68	0.19	0.30/1.05	<0.001
	Negative	−0.62	0.20	−1.02/−0.27	0.002
ADL (independent)	Dependence in ADL	−1.48	0.30	−2.07/−0.89	<0.001
Cognitive functioning (MMSE ≤ 24)	MMSE ≥ 25	1.00	0.30	0.41/1.59	<0.001
Depressive mood (no)	Yes	−7.84	0.35	−8.53/−7.16	<0.001
Number of morbidities (0)	1	−0.03	0.20	−0.35/0.41	0.881
	2	−0.19	0.23	−0.64/0.25	0.400
	≥3	−0.79	0.27	−1.32/−0.26	0.004
Time period of inclusion, years					
(Wave 1, 2001–2004)	Wave 2, 2006–2012	−0.60	0.22	−1.03/0.18	0.005
	Wave 3, 2012–2016	−1.65	0.26	−2.15/−1.14	<0.001
	Wave 4, 2017–2022	−0.44	0.24	−0.92/0.04	0.071
R^2^ = 0.106					

^1^ Mental Component Summary (MCS) from the 12-item Short Form Survey (SF-12). Depressive mood was excluded as an independent variable in the MCS analysis.

## Data Availability

The data that support the findings of this study are held by the Division of Geriatric Medicine, Lund University (PI Sölve Elmståhl), but restrictions apply to their availability. They were used under license for the present study and are not publicly available. However, the data are available from the authors upon reasonable request and with the permission of the Division of Geriatric Medicine, Lund University (PI Sölve Elmståhl).

## Abbreviations

ADL	Activities of Daily Living
FCS	fully conditional specification method
FOC	Fear of Crime
CPRS	Comprehensive Psychopathological Rating Scale
GÅS	the Good Aging in Skåne project
HRQoL	Health-Related Quality of Life
LS	Life Satisfaction
LSI-A	Quality of Life Scale A
MADRS	Montgomery-Åsberg Depression Rating Scale
MCS	Mental Component Summary
MMSE	Mini Mental State Examination
QoL	Quality of Life
PCS	Physical Component Summary
SF-12	Short form health survey
SNAC	Swedish National Aging and Care survey

## Appendix A

Table A1, Table A2 and Table A3 Sensitivity analysis examining the effect of multiple imputations on the adjusted associations between fear of crime and life satisfaction and quality of life related to physical and mental health.

**Table A1:** Multiple linear regression model with Neugarten’s LSI-A score as the dependent variable.

Fear of Crime (FOC)	LSI-A Life Satisfaction Index (LSI-A)			
Original Results ^1^ N = 5373	B	Robust Std. Error	95% CI	*p*-Value
	−0.54	0.21	−0.96/−0.12	0.011
	−1.54	0.32	−2.16/−0.92	<0.001
	−2.05	0.47	−2.97/−1.13	<0.001
Results after multiple imputations ^1^ N = 5832				
Occasionally	−0.58	0.21	−0.99/−0.17	0.006
Often	−1.42	0.30	−2.01/−0.82	<0.001
Very often	−1.96	0.46	−2.88/−1.05	<0.001

^1^ Adjusted for age, sex, education, cohabiting status, place of residence, financial status, alcohol habits, smoking habits, physical activity, ADL, cognitive status, attitude toward older adults, depressive mood,. disease categories and time of inclusion (waves 1–4).

**Table A2:** Multiple linear regression model with the SF-12 PCS score as the dependent variable.

Fear of Crime (FOC)	Health-Related Quality of Life Physical Component Summary (SF-12 PCS)			
Original Results ^1^ N = 5367	B	Robust Std. Error	95% CI	*p*-Value
	−0.93	0.25	−1.42/−0.44	<0.001
	−1.74	0.36	−2.45/−1.04	<0.001
	−2.43	0.58	−3.56/−1.30	<0.001
Results after multiple imputations ^1^ N = 5832				
Occasionally	−0.93	0.24	−1.41/−0.45	<0.001
Often	−1.43	0.35	−2.11/−0.75	<0.001
Very often	−2.25	0.54	−3.23/−1.18	<0.001

^1^ Adjusted for age, sex, education, cohabiting status, place of residence, financial status, alcohol habits, smoking habits, physical activity, ADL, cognitive status, attitude toward older adults, depressive mood, disease categories and time of inclusion (waves 1–4).

**Table A3:** Multiple linear regression model with the SF-12 MCS score as the dependent variable.

Fear of Crime (FOC)	Health-Related Quality of Life Mental Component Summary (SF-12 MCS)			
Original Results ^1^ N = 5519	B	Robust Std. Error	95% CI	*p*-Value
	−1.21	0.22	−1.63/−0.78	<0.001
	−1.89	0.32	−2.54/−1.26	<0.001
	−2.00	0.49	−2.95/−1.04	<0.001
Results after multiple imputations ^1^ N = 5832				
Occasionally	−1.18	0.22	−1.61/−0.75	<0.001
Often	−1.82	0.33	−2.47/−1.18	<0.001
Very often	−1.88	0.50	−2.86/−0.90	<0.001

^1^ Adjusted for age, sex, education, cohabiting status, place of residence, financial status, alcohol habits, smoking habits, physical activity, ADL, cognitive status, attitude toward older adults, disease categories and time of inclusion (waves 1–4).
